# Accuracy and Comfort in Digital and Conventional Impression in Pediatric Dental Patients: A Randomized Comparative Study

**DOI:** 10.7759/cureus.76882

**Published:** 2025-01-03

**Authors:** Chhaya Patel, Gunjan N Barot, Megha C Patel, Krati J Nath, Shruti P Patel, Dhruvi K Patel

**Affiliations:** 1 Department of Pedodontics and Preventive Dentistry, Karnavati School of Dentistry, Karnavati University, Gandhinagar, IND; 2 Department of Dentistry, Faculty of Dental Sciences, Dharmsinh Desai University, Nadiad, IND; 3 Department of Dentistry, Karnavati School of Dentistry, Karnavati University, Gandhinagar, IND

**Keywords:** alginate, clinical efficiency, digital impressions, dimensions, impression accuracy, pediatric dentistry

## Abstract

Background and aim: Pediatric dental impressions are a crucial component of dental care for children, and the choice between digital and conventional impression techniques can significantly impact the treatment outcomes and patient experience. Understanding the time efficiency and comfort levels associated with these techniques along with their accuracy is essential for informed decision-making in pediatric dentistry. This study aimed to compare digital and conventional impression techniques in pediatric dentistry, focusing on the accuracy of dimensions for either technique, time required, pain, gag reflex, and patient comfort.

Materials and method: A randomized crossover-controlled study was conducted with 23 pediatric patients aged eight to 12 years who required dental impressions. Patients were randomly assigned using computer-generated sequences to either the digital or conventional impression groups. In the first appointment, one group received the alginate impression first, while the other group had the digital scanner impression first. Then, in the second appointment, the crossover was performed and the sequence was reversed. The primary outcome evaluated was the accuracy of dimensions for either technique focusing on the mesiodistal width of central incisors, intercanine width, and intermolar width. Secondary outcomes recorded procedure duration, patient comfort, pain, and gag reflex using the visual analog scale (VAS). The data analysis was performed using SPSS software version 20.0 (Chicago, IL: IBM Corp.) at a significance level of p<0.05.

Results: Intercanine width measurements in both the maxilla and mandible showed no significant differences between scanner and alginate impressions. The intermolar width in the maxilla and the mesiodistal width of the permanent mandibular central incisor with the scanner gave higher measurements which were statistically significant (p=0.006 and p<0.001, respectively). Conventional alginate impressions resulted in significantly higher mean values for impression time, pain, and gag reflex (p<0.001). Children were found to be more comfortable with the intraoral scanning and it was statistically significant.

Conclusion: Digital intraoral scanning offers accuracy, speed, and improved patient comfort in pediatric dentistry, highlighting its potential to enhance diagnostic and treatment procedures and can serve as a valuable tool for specific clinical needs, balancing innovation and established practices.

## Introduction

Pediatric dentistry demands specialized techniques to ensure accurate and comfortable dental impressions, which play a pivotal role in the diagnosis, treatment planning, and fabrication of various dental appliances. Conventional impression techniques have long been the cornerstone of pediatric dentistry, offering clinicians tangible replicas of a patient's dentition [1]. Materials like alginate, appreciated for their cost-effectiveness and user-friendly nature, have been extensively utilized in capturing primary and mixed dentition. Traditional methods, though reliable, have inherent limitations such as distortion, extended setting time, and discomfort, particularly for pediatric patients. Their sensitivity to stimuli can exacerbate anxiety and gag reflexes during impression procedures and can hamper cooperation from young children. This necessitates an exploration of alternative impression techniques that not only address the technical limitations but also prioritize the comfort and cooperation of young patients undergoing dental procedures.

The emergence of digital impression techniques has introduced cutting-edge technologies, such as intraoral scanners and three-dimensional (3D) imaging, to the realm of pediatric dentistry [2]. Digital impressions offer several advantages over their conventional counterparts. Firstly, the elimination of physical materials reduces the potential for distortion and inaccuracies. Secondly, the digital workflow allows for real-time visualization of the scanned area, enabling immediate adjustments and retakes if necessary. These features contribute to a more comfortable experience for pediatric patients, as digital impressions are often quicker and less invasive compared to traditional methods.

In addition to improved accuracy of impression and patient comfort, digital impression techniques facilitate seamless integration with computer-aided design and manufacturing (CAD/CAM) systems [3]. This integration streamlines the fabrication process of dental appliances, crowns, and other restorations, enhancing overall treatment efficiency. Furthermore, the digital data captured can be easily stored, shared, and retrieved, contributing to enhanced communication among dental professionals and interdisciplinary collaborations. Various manufacturing companies have been producing intraoral scanners, each offering unique advantages to practitioners. These advantages include the size of the wand, use of powder, scanning method, ability to capture color and record a full mouth scan, ease of using software, device portability, time required for scanning, and the compactness of the scanner. The intraoral scanners differ in image acquisition methods, unit size, speed, and weight. Techniques such as triangulation, active wavefront sampling (AWS), parallel confocal system, accordion fringe interferometry (AFI), three-dimensional in-motion video, telecentric system, and intraoral scanner (IOS) fast scan have been introduced which can affect the accuracy and outcomes. Thus it is crucial to check the efficacy of every available scanner due to the varying parameters in size, speed, weight, and image acquisition methods, ensuring optimal performance and accuracy for specific clinical needs [4].

Currently, marketed intraoral scanning systems include the iTero Element (San Jose, CA: Align Technology), TRIOS 3 (Copenhagen, Denmark: 3Shape), True Definition (St. Paul, MN: True Definition), Cerec AC OmniCam (Bensheim, Germany: Dentsply Sirona), CS3600 (Atlanta, GA: Carestream Dental), PlanScan (Helsinki, Finland: Planmeca), and Medit (Seoul, South Korea: Medit Corporation) [5]. In the present study, we have used the Irific intraoral scanner (Mumbai, India: NeuralHive), which is claimed by the company to be highly accurate, saving time with a one-minute scan and providing a better patient experience by avoiding uncomfortable impression trays. The scanner produces precise margins, leading to better-fitting prostheses and longer-lasting restorations. It offers virtual articulation for accurate bite registrations and removes undercuts, saving chair-side time. Automated shade selection ensures predictable esthetic results and impressions can be sent electronically to the lab, reducing recurring costs and facilitating digital orthodontics, including clear aligner treatments. As fewer studies have been done on it, the scope for newer evaluations for potential advancements increases.

Despite the evident benefits of digital intraoral scanning, it is crucial to acknowledge that both conventional and digital methods have their respective merits and limitations. Factors such as accuracy of impression, cost, accessibility, and the learning curve associated with adopting new technologies influence the choice between the two approaches [6]. The selection of an impression technique in pediatric dentistry should be based on a comprehensive evaluation of the specific clinical scenario, considering the child's age, behavior, and the nature of the dental procedure. As the field advances, it is crucial to rigorously evaluate the comparative effectiveness of digital versus traditional impression methods in pediatric dentistry.

This study aimed to contribute valuable insights into the practical implications, outcomes, and patient experiences associated with both approaches. The null hypothesis proposed was that there is no significant difference in the dimensions, as well as in the time, pain, gag reflex, and patient comfort between the conventional and digital intraoral scanning impression procedures. By exploring and understanding the nuances of each technique, pediatric dentists can make informed decisions that prioritize the well-being of their young patients while optimizing treatment outcomes.

## Materials and methods

Study design and ethical approval

This study followed a prospective, randomized crossover parallel arm design to assess and compare two impression techniques - conventional alginate and digital intraoral scanning - in pediatric dental patients aged eight to 12 years. The study received ethical approval from the ethics committee to uphold ethical standards. The registration number is KSDEC/23-24/Apr/017. Informed consent was obtained from the parents or legal guardians of each participating child to ensure transparency and compliance with ethical guidelines.

Participants

Conducted at the Department of Pediatric and Preventive Dentistry, Karnavati School of Dentistry, Gujarat, India, this comprehensive study focused on a diverse cohort of children aged eight to 12 years. The inclusion criteria meticulously defined the age range as well as fully erupted permanent first molars, permanent mandibular central incisors, and presence of deciduous maxillary and mandibular canine which were caries-free, not restored, and without any anomalies to ensure a targeted examination of pediatric dental impressions in a critical developmental period. To maintain the study's precision, exclusion criteria were thoughtfully established, encompassing factors such as non-compliance, syndromes, systemic diseases, developmental anomalies, significant dental trauma, known allergies to impression materials used in the study, cleft lip and palate conditions and inadequate proficiency in the language used for communication, hindering comprehension of study procedures and instructions.

Sample size determination and randomization

Considering a null hypothesis for the proportion of 50% between the two methods, with a 5% alpha error, 90% study power, and a clinically significant difference of two units, the sample size was calculated at 23 participants. In this experimental design, participants were divided into two groups to test the following two impression techniques: conventional alginate (group 1) and digital intraoral scanning (group 2). Block randomization was employed to ensure balanced group sizes, with the random sequence generated using computer-generated random numbers. Allocation concealment was maintained by having an independent researcher, not involved in data collection or analysis, and by preparing sealed, opaque envelopes containing the group assignments.

**Figure 1:**
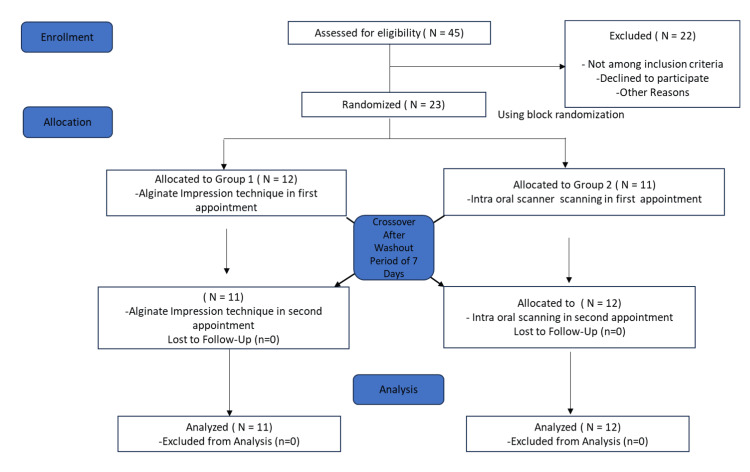
Consolidated Standards of Reporting Trials (CONSORT) diagram. The image is created by the author, Gunjan Barot, of this study.

Additionally, secondary randomization was conducted to determine whether the maxilla or mandible would be measured first. This sequence was also generated using computer-based randomization, with the assignments placed in sealed envelopes to ensure blinding. These measures minimized selection bias and enhanced the methodological rigor of the study (Figure 1).

**Figure 2:**
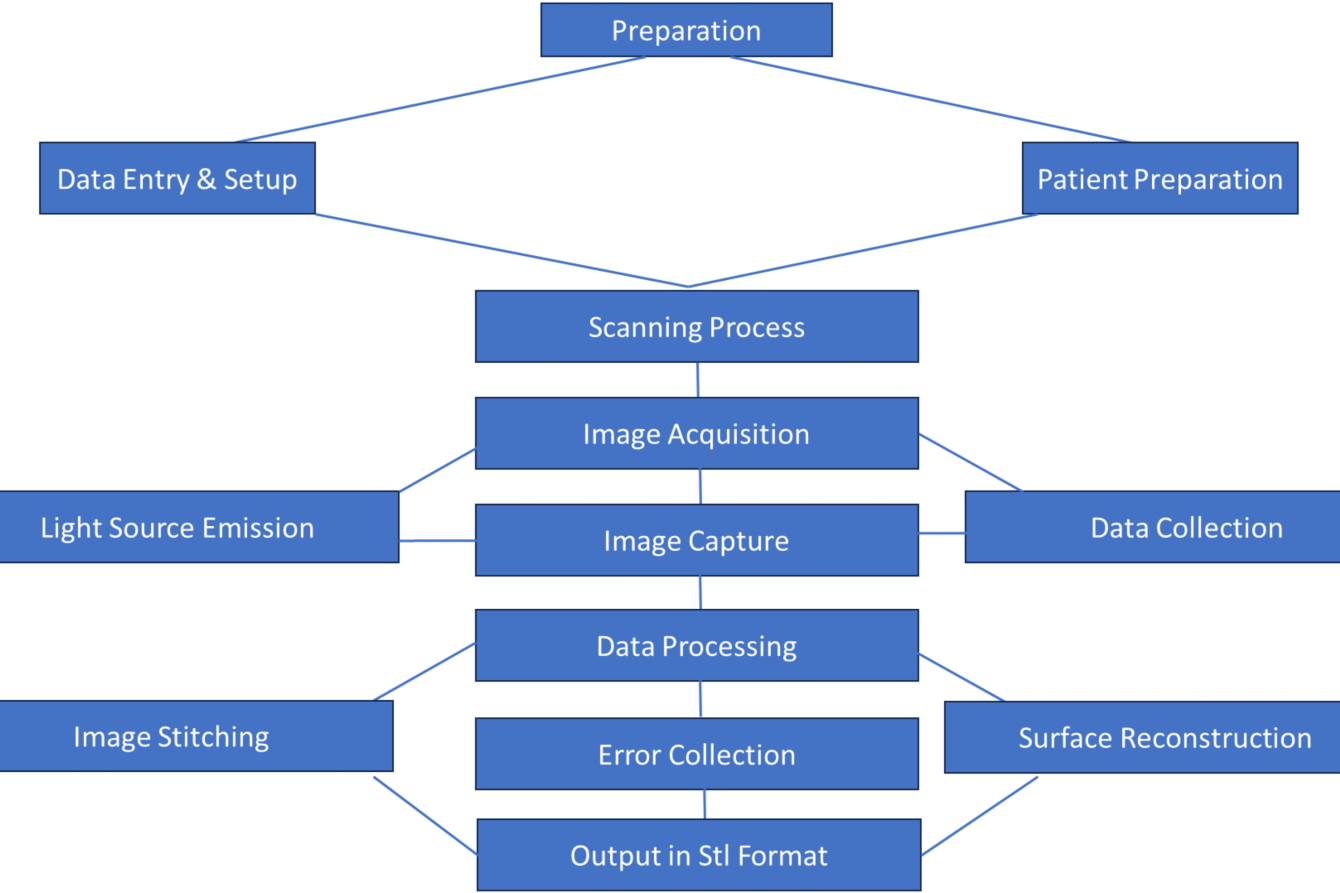
Workflow of the scanning process. The image is created by the author, Gunjan Barot, of this study.

During the initial appointment, participants in group 1 underwent the alginate impression technique first, while those in group 2 experienced the digital scanner impression technique. The detailed workflow for the scanning process, from preparation to data processing and output, is depicted in Figure 2.

After this, a one-week washout period was observed. In the subsequent appointment, a crossover was performed and the other impression technique was employed. This crossover design ensured a balanced distribution and minimized potential bias stemming from the order of the impression techniques.

Operator and procedures

Alginate Impression

A skilled operator with extensive experience in pediatric dentistry and trained in both techniques performed all impressions to ensure standardization and minimize variability. To maintain intraoperator reliability, the operator underwent calibration sessions prior to the study. These sessions involved taking multiple alginate impressions on dental models and real subjects under conditions similar to those of the study, with each impression being evaluated for accuracy and consistency by a supervising expert.

The conventional impression was taken using Impreceed Alginate Impression Material (Tokyo, Japan: GC Corporation), with a total working time of 90 seconds. The alginate material was mixed by hand with tap water, using a 1:1 water-to-powder ratio measured with a cylindrical cup for water and a measuring scoop for powder. Mixing was performed rapidly with a figure-eight motion against the side of a rubber bowl using a wide-blade spatula, resulting in a creamy consistency that did not fall from the spatula when lifted. This was then loaded into a properly sized impression tray for taking the impression. The time (in seconds) was measured with a stopwatch (Kardiff Racer; Yiwu, China: Shanghai Kuke Sport Co., Ltd.) from the start of mixing until the impression was taken out. To further reduce variability, the operator adhered strictly to a standardized protocol for all steps, including tray selection, mixing consistency, and impression removal. The impressions were poured into type III gypsum products (Kaldent; Mumbai, India: Kalabhai Karson Pvt. Ltd) mixed on a vibrator to avoid porosities. Bases were made with type II gypsum products (Kaldent; Mumbai, India: Kalabhai Karson Pvt. Ltd).

Digital Scanning

**Figure 3:**
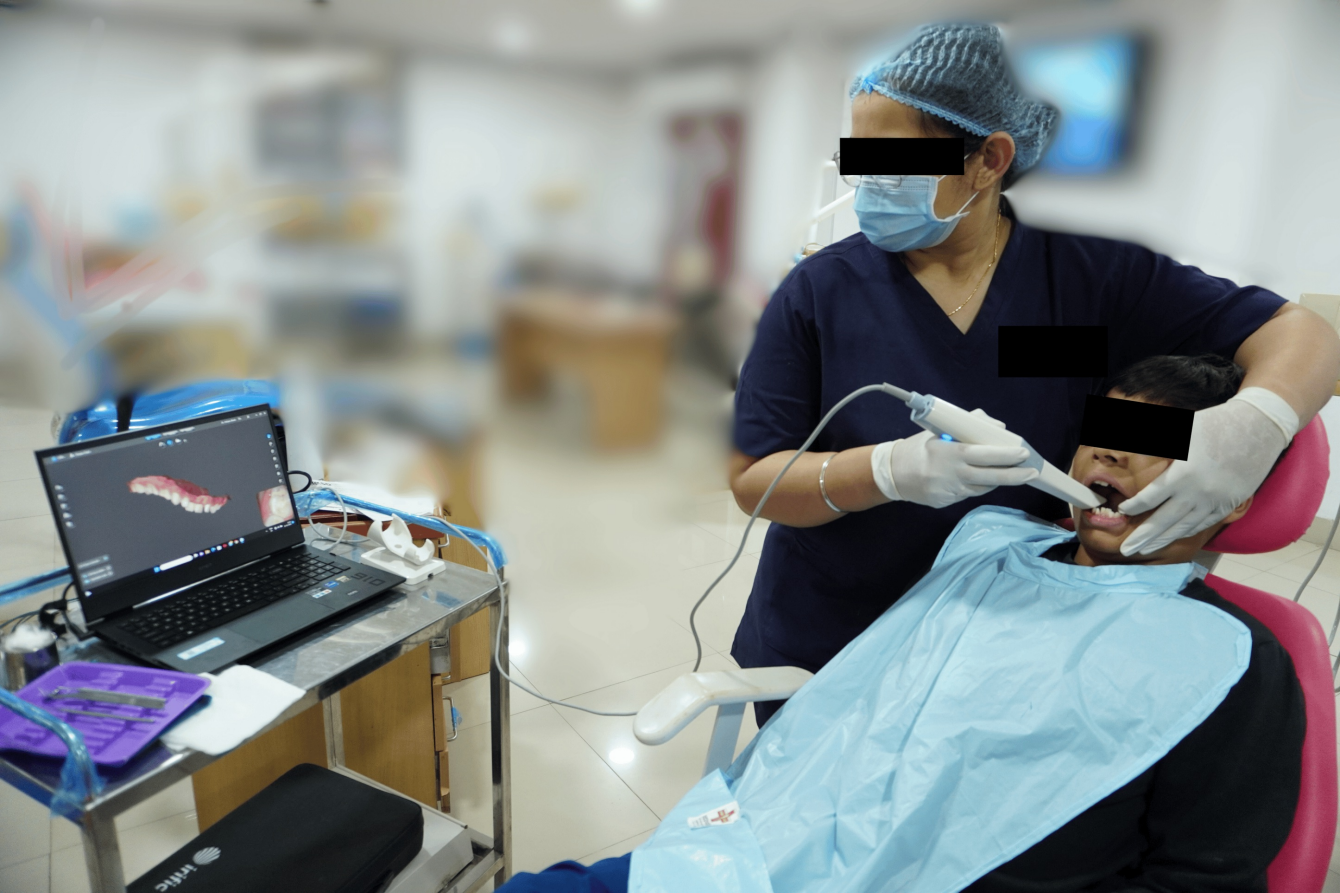
Intraoral scanning procedure in pediatric patient. The image is captured by the author, Gunjan Barot, of this study.

The operator utilized the Irific intraoral scanner (Mumbai, India: NeuralHive) for digital impressions, strictly adhering to the manufacturer's recommended protocol. Scanning time was measured using the scanning software. The scanning process began with the sequential scanning of the occlusal, buccal, and lingual surfaces of both arches, concluding with bite registration (Figure 3).

Parameters assessed

The study evaluated the precision of dimension measurements using two different techniques, focusing on key parameters such as the mesiodistal width of permanent mandibular central incisors, intercanine width, and intermolar width. Secondary evaluation criteria included the duration of the impression-making process, measured in seconds using a stopwatch. Pain during the impression-taking, gag reflex, and patient comfort were assessed post-session using a visual analog scale (VAS) ranging from 0 to 10.

**Figure 4:**
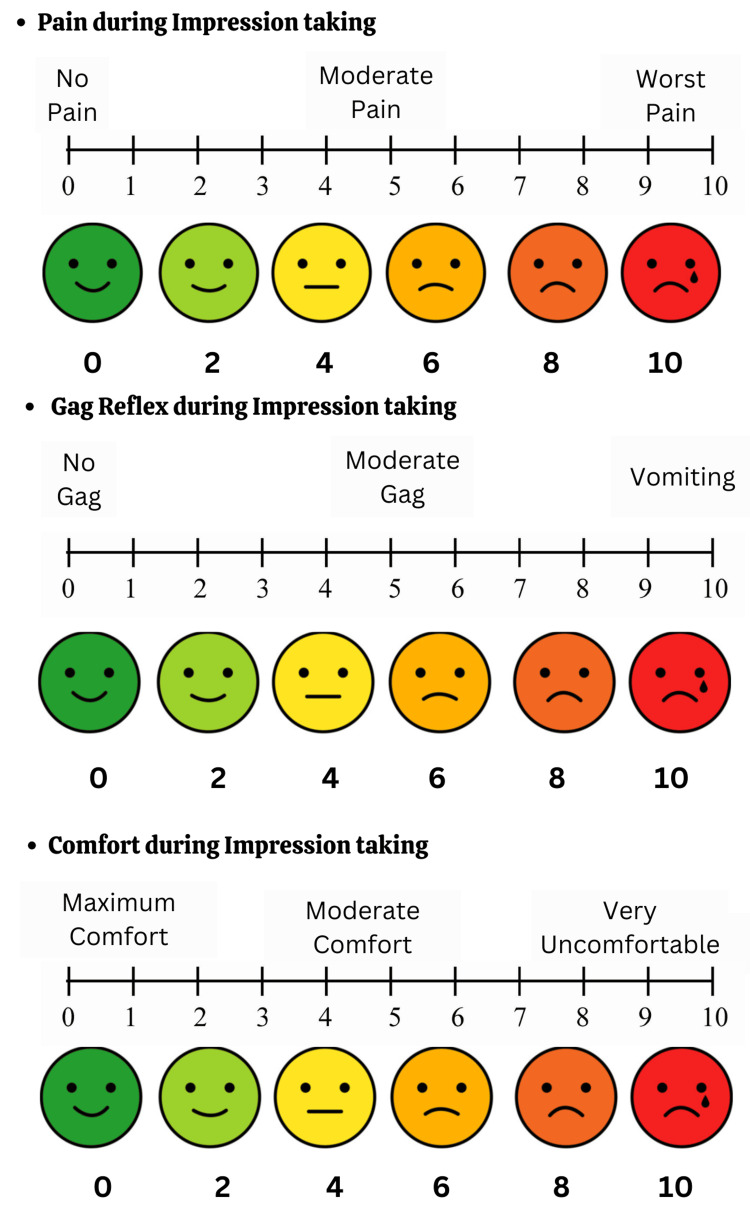
Visual analog scale (VAS) scale. The image is created by the author, Gunjan Barot, of this study.

To ensure accurate VAS assessment, the scale was explained to the pediatric patients in an age-appropriate manner. The children were shown the image in Figure 4. A simple verbal explanation and demonstration helped them understand how to use the scale. After each impression, patients were asked to mark the point on the line that best represented their experience regarding pain, gag reflex, and overall comfort during the procedure. This method allowed for consistent and reliable data collection.

**Figure 5:**
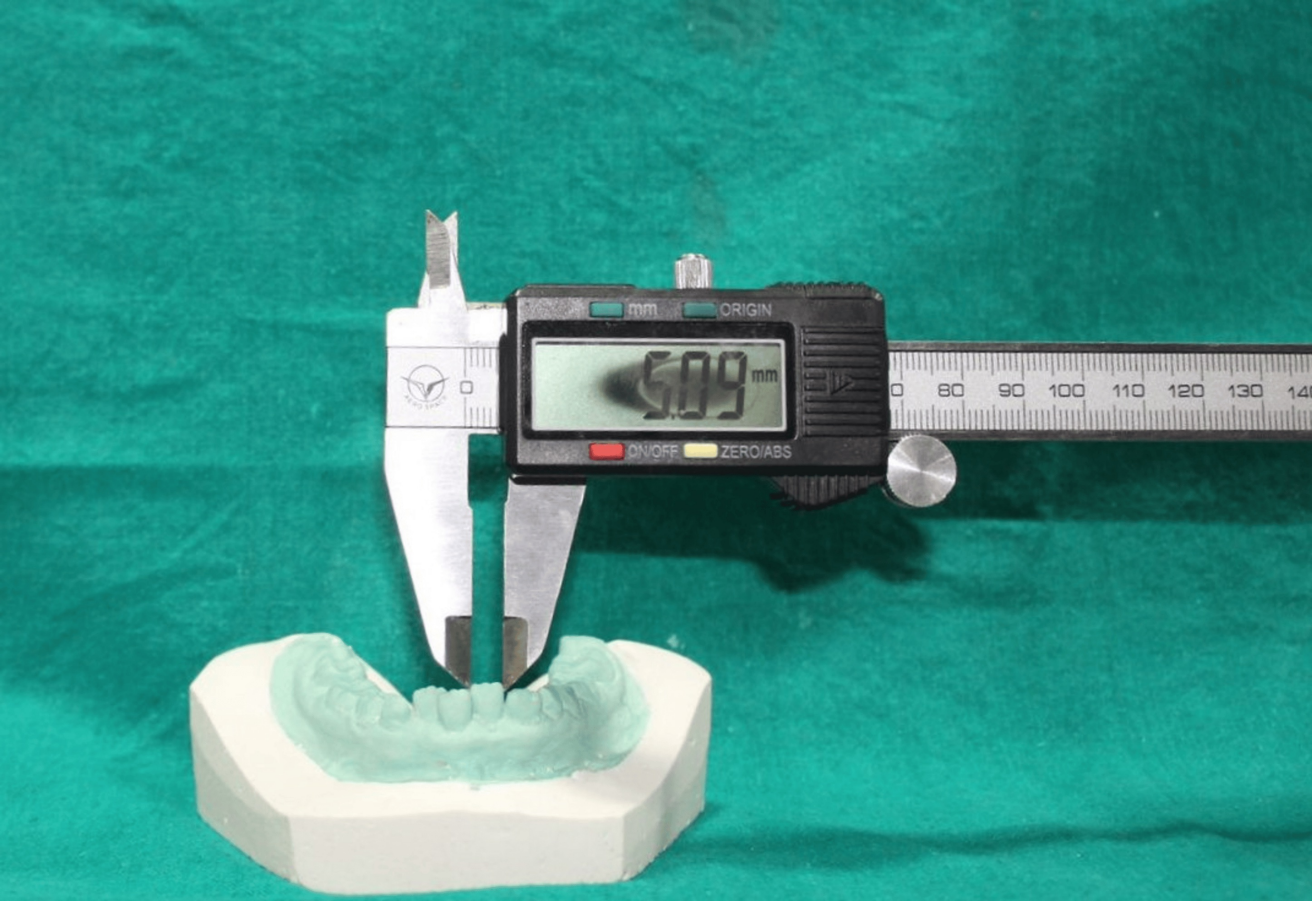
Measurement of mesiodistal width of permanent central incisor with vernier caliper. The image is captured by the author, Gunjan Barot, of this study.

**Figure 6:**
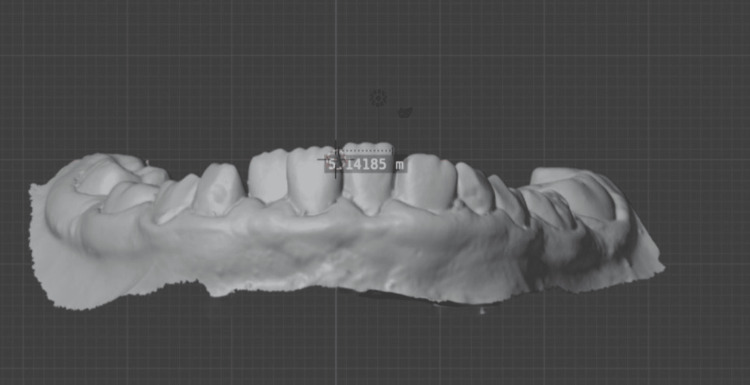
Measurement of mesiodistal width of permanent central incisor digitally. The image is generated by the author, Gunjan Barot, of this study.

In this study, validity is considered to be the extent to which the measurements from the digital system agreed with the caliper measurements. Dimensional measurements were conducted using vernier calipers on the cast poured from an impression (Figure 5) and digitally in the intraoral scanner via the software ruler (Blender 4.1 software; Amsterdam, Netherlands: Blender Foundation), with specific reference points as mentioned further (Figure 6).

The permanent mandibular central incisor width is measured from the crest of curvatures on the mesial surface to the distal surface. The intercanine width is defined as the distance between the cusp tips of the right and left maxillary and mandibular deciduous canines. The intermolar width is assessed as the distance between the mesiobuccal cusp tips of both the right and left maxillary and mandibular permanent first molars.

Data collection and analysis

A Microsoft Excel (Microsoft Office 2019; Redmond, WA: Microsoft Corp.) spreadsheet was used to enter the data and any inconsistencies were examined. The Shapiro-Wilk test was employed to verify if every variable adhered to a normal distribution. Statistical analysis, encompassing descriptive statistics and inferential tests, was executed to compare outcomes between the two impression techniques. Data analysis was done by SPSS software version 20.0 (Chigaco, IL: IBM Corp.), and parametric tests such as Pearson's test and the paired t-test were used for bivariate analysis at a 5% significance level.

## Results

There were no significant differences in intercanine width measurements between the scanner and alginate techniques in both the maxilla and mandible. However, in both the maxilla and mandible, the scanner showed a greater intercanine width compared to the conventional method, with mean differences of 0.11 and 0.07, respectively. These findings suggest that both impression techniques provide comparable intercanine width measurements (Table 1).

**Table 1:** Intragroup comparison of intercanine width between alginate and scanner group. *Results are expressed as mean±SD, with the mean difference and significance level analyzed using a paired t-test. P<0.005 was considered significant.

Variable		Group	Mean±SD (mm)	Mean difference±SD (mm)	t-Value	p-Value*
Intercanine width (mm)	Maxilla	Scanner	31.42±1.63	0.11±0.49	0.22	0.102
		Alginate	31.31±1.71			
	Mandible	Scanner	25.33±1.95	0.07±0.18	1.74	0.096
		Alginate	25.26±1.94			

For the intermolar width, the results show a significant difference between the scanner and alginate techniques in the maxilla but not in the mandible. In the maxilla, there was a significant increase of 0.07 mm (p=0.006) in the scanner group. While in the mandible, both techniques had similar measurements (Table 2). For the mesiodistal width of the central incisor, the findings revealed a significant difference between the scanner and alginate techniques, with the scanner showing a statistically higher mean difference of 0.1±0.09 mm (Table 3).

**Table 2:** Intragroup comparison of intermolar width between alginate and scanner group. *Results are presented as mean±SD, mean difference, and p-value determined using a paired t-test. P<0.005 was considered significant.

Variable		Group	Mean±SD (mm)	Mean difference±SD (mm)	t-Value	Significance level
Intermolar width (mm)	Maxilla	Scanner	48.3±2.79	0.07±0.12	3.01	0.006
		Alginate	48.23±2.75			
	Mandible	Scanner	40.63±1.95	0±0.37	0.00	1
		Alginate	40.62±1.94			

**Table 3:** Intragroup comparison of mesiodistal width of central incisor between alginate and scanner group. *Statistical analysis was conducted using a paired t-test to determine the p-value. P<0.005 was considered significant.

Variable	Group	Mean±SD (mm)	Mean difference±SD (mm)	t-Value	p-Value*
Mesiodistal width of central incisor (mm)	Scanner	5.25±0.34	0.16±0.09	5.62	<0.001
	Alginate	5.09±0.31			

Upon analysis, significant differences emerged between the two impression procedures across several secondary key parameters. Notably, conventional alginate impressions exhibited markedly higher mean values for scanning time, pain, gag reflex, and comfort when compared to digital intraoral scanning impressions. Specifically, participants undergoing conventional alginate impressions experienced a mean time difference of 187 seconds longer compared to digital intraoral scanning. Moreover, pain levels were reported to be higher in the alginate group, while the gag reflex parameter was also greater in the alginate group. Patients reported a higher level of comfort with the scanner, with a significance level of p<0.001 (Table 4).

**Table 4:** Intragroup comparison of secondary outcomes between alginate and scanner group. *Mean±SD values are presented alongside the mean difference and significance level, determined using a paired t-test. P<0.005 was considered significant.

Variable	Group	Mean±SD (mm)	Mean difference±SD (mm)	t-Value	Significance level
Time (s)	Scanner	223.91±36.64	-187±40.3	-21.07	<0.001
	Alginate	410.91±15.02			
Pain	Scanner	3.57±1.44	-2.26±1.39	-7.81	<0.001
	Alginate	5.83±1.3			
Gag reflex	Scanner	3.13±1.22	-3.48±0.9	-18.58	<0.001
	Alginate	6.61±1.2			
Comfort	Scanner	4.04±1.02	-2.09±1.35	7.44	<0.001
	Alginate	6.13±1.06			

In Pearson’s correlation test, all parameters showed a strong positive correlation. The findings revealed strong positive correlations between intercanine width measurements in both the maxilla (r=0.988, p<0.001) and mandible (r=0.996, p<0.001), mesiodistal width of the central incisor (r=0.969, p<0.001), and intermolar width measurements in the maxilla (r=0.999, p<0.001) and mandible (r=0.981, p<0.001), indicating consistent anatomical measurements. Also, a significant correlation was found for the time as well as the comfort parameter. Pain levels showed a moderate positive correlation while gag reflex responses exhibited a strong positive correlation between the two techniques (Table 5).

**Table 5:** Pearson’s correlation test for alginate and scanner groups. *Correlation value. **The p-values were determined using Pearson's correlation test. N: number of participants. P≤0.005 was considered significant.

S. no.	Parameters being correlated	N	R-value*	p-Value**
1	Time (seconds) for scanner vs. time (seconds) for alginate	23	-0.051	<0.001
2	Intercanine width in the maxilla (scanner vs. alginate)	23	0.988	<0.001
3	Intercanine width in the mandible (scanner vs. alginate)	23	0.996	<0.001
4	Mandibular (Md) width of canine teeth (scanner vs. alginate)	23	0.969	<0.001
5	Intermolar width in the maxilla (scanner vs. alginate)	23	0.999	<0.001
6	Intermolar width in the mandible (scanner vs. alginate)	23	0.981	<0.001
7	Pain level with scanner vs. pain level with alginate	23	0.491	<0.01
8	Gag reflex with scanner vs. gag reflex with alginate	23	0.723	<0.001
9	Comfort level with scanner vs. comfort level with alginate	23	0.163	<0.001

## Discussion

Pediatric dentistry has been related to anxiety for ages. Impression techniques can vary the anxiety response of pediatric patients. The present study aimed to compare the efficacy and outcomes of conventional alginate impressions versus digital intraoral scanning impressions in pediatric dental patients. Our findings offer valuable insights into the comparative performance of these impression techniques across various parameters.

In our study, we observed minimal differences in the dimensions of the evaluated parameters. It can be noted that intercanine width showed a non-significant mean difference when evaluated by either technique, while intermolar width in the maxilla and mesiodistal width of permanent central incisors had statistically higher differences in the digital scanner group. Caliper measurements in this study served as the standard for comparison with other measuring methods. Hayashi et al. examined the accuracy and reliability of the Sure Smile Ora Scanner (Charlotte, NC: Dentsply Sirona), Vivid910 Scanner (Copenhagen, Denmark: 3Shape), and R700 Scanner (Hangzhou, China: Shining 3D), and they reported all scanners to be sufficiently accurate compared to the vernier caliper and found no significant difference in reliability between the comparisons [7]. Similar results were observed by Murugesan and Sivakumar [8]. It is worth noting that while neither method perfectly reproduces intraoral structures, conventional alginate impressions may offer a more representative depiction due to their ability to capture intricate detailing. Importantly, the trueness of both impression techniques was found to be similar in our study which is consistent with a recently published systematic review by Kong et al. [9].

The significant difference in dimensions between alginate and scanner groups in our study may have resulted from the use of third-party measurement software, as the specific scanner program did not include a calibration ruler, which could also be a possible factor. However, contrasting findings were reported by Tomita et al., who suggested that direct intraoral scanning might be more accurate than conventional study models. They found that measurements calculated from both direct and indirect scanning methods demonstrated high accuracy suitable for clinical practice [10].

In the present study, a noteworthy observation was made regarding the time required for impression taking, which was found to be longer when using alginate compared to intraoral digital scanning. The extended duration needed for alginate impressions in contrast to intraoral digital scanning can be attributed to various factors, including the preparation of materials, the time required for the material to be set, considerations for patient comfort, adjustments needed for tray fitting, and the cleanup process after impression-taking. In the present study, we measured the time from the start of mixing alginate, which could explain the longer duration observed in the alginate group. Understanding the implications of impression time, whether with conventional alginate or digital scanning, is crucial for optimizing patient comfort, procedural efficiency, and overall treatment outcomes. While Yilmaz and Aydin found no significant difference in total impression time between the two procedures [11], Burhardt et al. and Mangano et al. reported significantly shorter times for conventional alginate impressions [12,13]. Also, Rolfsen et al. showed a shorter time for alginate impressions which could be attributed to the fact that impressions were taken over a typhodont and not in patients [1]. In a study by Schepke et al., the digital impression method was found to be more efficient when evaluating the total treatment time [14]. The digital system required fewer repetitions, which helped reduce the overall treatment time. Additionally, in the study by Asquith et al., inexperienced operators found the digital technique less challenging compared to the conventional impression method [15]. This aligns with the findings of the present study.

A notable difference was observed favoring the scanner group over the alginate group in terms of comfort and gag reflex, with statistical significance in the present study. Alginate impressions may trigger discomfort due to their odor, texture, and bulk, potentially contributing to gag reflexes. Additionally, issues like oversized trays or extensions and subjective pressure during the impression process can lead to perceived pain, factors largely absent in intraoral scanning procedures. Also, only successful impressions were included in the study, highlighting the increased time required for alginate impressions in uncooperative patients compared to digital scans, which required no repetitions, and hence the comfort also increased. These findings are consistent with previous studies by Burdaht et al. and Mangano et al. that also reported similar outcomes for these variables [12,13]. Moreover, no significant difference between the two procedures regarding pain was observed, a result echoed in another study conducted by Yilmaz and Aydin [11].

Intraoral scanners, despite their advancements, present several limitations. These include high costs associated with acquisition and maintenance, a learning curve for dental professionals transitioning from traditional methods, limited accessibility due to device design constraints, compatibility issues with software and hardware systems, and the need for regular maintenance and updates. Addressing these limitations is essential to maximize the benefits of intraoral scanning technology while ensuring efficient and effective dental practice, acknowledging the enduring utility and reliability of conventional alginate impressions in various clinical scenarios.

Strength of study

The randomized crossover design employed in this study significantly enhances the reliability of its findings. By minimizing intersubject variability and including a single trained operator for both the scanner and alginate procedures, potential operator-related biases are further eliminated, ensuring consistency and reducing confounding factors. This methodological approach strengthens the study's internal validity, allowing for more accurate comparisons between the two impression techniques without the influence of differing operator skills.

Limitations

The study had several limitations. The sample size was relatively small, which could affect the generalizability of the results. Additionally, the research was limited to a single age group (8-12 years), potentially restricting its applicability to other pediatric age groups. The findings were also influenced by the subjective responses of participants, which may include some degree of bias, despite efforts to standardize the evaluation process. Furthermore, the use of specific software and devices in the study might limit its reproducibility using other systems.

## Conclusions

In summary, our study implies the distinct advantages of digital intraoral scanning over conventional alginate impressions in pediatric dentistry. Notably, we found that digital scanning offers expedited impression-taking processes with optimal accuracy in dimensions and greater patient comfort, particularly in reducing gag reflexes. These findings shed light on the transformative potential of digital technology in improving dental procedures, albeit with considerations like cost and learning curve. While digital scanning presents promising advancements, it is crucial to recognize the enduring utility of traditional alginate impressions in dentistry. Thus, this study contributes to the evolving landscape of dental practice by offering insights into optimizing patient care through innovative technologies while acknowledging the importance of traditional methods in dental care.
